# Gene Expression Profiles of Inflammatory Mediators in Influenza A and B Virus Infections: Insights from Riyadh, Saudi Arabia (2020–2023)

**DOI:** 10.3390/genes17030325

**Published:** 2026-03-17

**Authors:** Noorah A. Alkubaisi, Mohamed A. Farrag, Ibrahim M. Aziz, Reem M. Aljowaie, Fahad N. Almajhdi

**Affiliations:** Department of Botany and Microbiology, College of Science, King Saud University, P.O. Box 2455, Riyadh 11451, Saudi Arabia; nalkubaisi@ksu.edu.sa (N.A.A.); iaziz@ksu.edu.sa (I.M.A.); raljowaie@ksu.edu.sa (R.M.A.); vrg_ksu@yahoo.com (F.N.A.)

**Keywords:** influenza viruses, cytokines, chemokines, gene expression

## Abstract

Background/Objectives: *Influenza A* (IAV) and *influenza B* (IBV) viruses pose significant public health threats, with varying epidemiology and immune responses. Limited subtype-specific cytokine data exist for influenza in Saudi Arabia. This study conducted molecular surveillance on 380 NPAs from patients at King Khalid University Hospital (KKUH) in Riyadh, Saudi Arabia, during winter seasons (2020–2023). Methods: NPA samples were collected from hospitalized patients presenting with fever (>38 °C) and respiratory symptoms. RNA was extracted using the QIAamp Viral RNA Kit, followed by RT-PCR for IAV (H1N1, A/H3N2) and IBV detection. Quantitative real-time PCR profiled mRNA expression of 17 cytokines/chemokines in IAV-positive (*n* = 65) and IBV-positive (*n* = 20) samples, normalized to *GAPDH* using the 2^−ΔΔCq^ method. Appropriate statistical tests were applied (*p* < 0.05 significant). Results: Results showed 17.11% IAV positivity (7.89% A/H1N1, 9.21% A/H3N2) and 5.26% IBV. A/H3N2 predominated, increasing from 6.67% (2020/21) to 12.30% (2022/23). Males had higher IAV rates (25.88% vs. 10.00% females, *p* < 0.05), while IBV was higher in females (6.67% vs. 3.53%). Age-wise, 0–4 years had peak IAV (28.42%, *p* < 0.05); IBV peaked at 5–14 years (10.91%). IAV elicited higher mRNA expression *IFN-α*, *IL-10*, *IL-13*, and *CCL-2* (*p* < 0.05); IBV showed elevated *IL-1α*, *IL-6*, and *IL-33* (*p* < 0.05). Within IAV, A/H1N1 had higher *IL-4*, *IL-10*, *IL-13*, and *IL-17*; A/H3N2 elevated *TNF-α*, *IL-6*, *IL-22*, *CCL-3*, and *CCL-4* (*p* < 0.05). Conclusions: These findings highlight subtype-specific inflammatory profiles and demographic disparities in Saudi Arabia, informing targeted interventions. Post-COVID resurgence underscores surveillance needs amid travel and gatherings. Insights into cytokine dynamics aid prognosis and therapeutics, emphasizing regional molecular monitoring for vaccine optimization and outbreak prevention.

## 1. Introduction

Influenza viruses represent a significant global public health challenge due to their ability to cause seasonal epidemics and occasional pandemics. IAV and IBV are the primary causes behind annual human infections, with IAV further subdivided into subtypes such as A/H1N1 and A/H3N2 based on hemagglutinin (HA) and neuraminidase (NA) surface glycoproteins. These viruses exhibit high genetic variability through antigenic drift and shift, enabling them to evade host immunity and necessitate annual vaccine updates [1,2]. Globally, seasonal influenza leads to an estimated 3–5 million severe cases and 290,000–650,000 deaths annually, disproportionately affecting vulnerable populations including children, the elderly, and those with comorbidities [3,4]. In regions with diverse climates like the Middle East, including Saudi Arabia, influenza circulation often peaks during winter months, exacerbated by factors such as international travel and mass gatherings like Hajj pilgrimages [5,6]. Molecular surveillance is crucial for tracking viral evolution, identifying emerging strains, and informing vaccination strategies [7,8,9,10]. Recent studies highlight the co-circulation of IAV subtypes and IBV lineages, with A/H3N2 often predominating in certain seasons due to its rapid mutation rate [9,11,12].

The economic burden is substantial, with healthcare costs and productivity losses amounting to billions worldwide [13,14,15]. In Saudi Arabia, local epidemiology shows varying prevalence, with A/H3N2 and A/H1N1 strains commonly detected in clinical samples from Riyadh and surrounding areas [8,16,17]. Understanding regional patterns is essential for tailored interventions [6]. Furthermore, the interplay between viral genetics and host factors influences disease severity [18,19,20]. Advances in whole-genome sequencing have revealed clade shifts, such as from 6B.1 to 5a.2a in Saudi isolates, underscoring the need for ongoing monitoring [8,16]. Inflammatory mediators play a pivotal role in pathogenesis, amplifying immune responses that can lead to cytokine storms in severe cases [21,22].

The inflammatory response to influenza infection is a double-edged sword, essential for viral clearance but potentially detrimental when dysregulated. Upon infection, IAV and IBV trigger innate immune pathways via pattern recognition receptors like Toll-like receptors (TLRs), leading to the production of pro-inflammatory mediators (e.g., IL-6) [18,23]. These mediators recruit immune cells and enhance adaptive immunity but can escalate to hyperinflammation, contributing to acute respiratory distress syndrome (ARDS) and multi-organ failure in severe cases [18,24]. Studies indicate distinct cytokine profiles between IAV subtypes; for instance, A/H3N2 infections often elicit stronger pro-inflammatory responses, correlating with increased disease severity, while A/H1N1 and overall IAV tend toward regulatory/Th2-biased patterns [19,22]. IBV, while generally milder, induces unique patterns with elevated IL-1α and IL-33, reflecting differences in viral-host interactions [25,26]. In ex vivo models, donor-specific factors such as age, smoking, and comorbidities modulate cytokine output; for example, diabetes negatively impacts IL-6 and TNF-α levels during A/H3N2 infection [18,27].

Differences between IAV and IBV extend beyond epidemiology to molecular and immunological profiles, influencing clinical outcomes. IAV’s broader host range and reassortment potential make it more prone to pandemics, as seen with the 2009 A/H1N1 outbreak, while IBV is human-specific with two lineages (Victoria and Yamagata) that co-circulate or alternate dominance [28,29]. In terms of inflammatory mediators, IAV infections are typically characterized by a regulatory/Th2-oriented profile with prominent antiviral elements, whereas IBV elicits a more pro-inflammatory and alarmin-driven signature. Subtype-specific variations within IAV are notable; A/H1N1 shows greater Th2/Th17 polarization, while A/H3N2 is associated with enhanced pro-inflammatory and chemotactic responses [18,19,22,26]. These profiles correlate with demographics: young children (0–4 years) show higher IAV positivity, while IBV peaks in 5–14-year-olds [11,21]. Genetic diversity, including unique glycosylation sites in HA, facilitates immune evasion and vaccine mismatch [11,16]. Recent reviews emphasize the role of bibliometric analyses in identifying hotspots like cytokine storms in influenza research [12,21]. Therapeutic updates focus on universal vaccines targeting conserved epitopes to address drift [30,31]. Understanding these distinctions is vital for predicting severity and optimizing management [29,32].

In Saudi Arabia, gaps persist in Saudi-specific data on post-pandemic cytokine and chemokine profiles in IAV and IBV infections. Limited subtype-specific cytokine data exist for influenza in Saudi Arabia, particularly regarding post-pandemic inflammatory profiles in IAV (A/H1N1 vs. A/H3N2) and IBV infections in urban centers like Riyadh. This study addresses these gaps by analyzing 380-NPAs from Riyadh patients (2020–2023) to delineate prevalence, demographic patterns, and cytokine/chemokine profiles in IAV (A/H1N1, A/H3N2) and IBV infections. Therefore, the primary objectives are: (1) to determine the prevalence and seasonal distribution of IAV subtypes and IBV; (2) to identify demographic (age, gender) associations with infection rates; (3) to profile inflammatory mediators using qPCR and compare signatures between IAV/IBV and IAV subtypes; and (4) to inform regional surveillance and therapeutic strategies for improved influenza management in Saudi Arabia. By employing RT-PCR for detection and qPCR for mediator quantification, we address gaps in regional data, particularly post-pandemic shifts. These descriptive findings on subtype-specific mRNA expression patterns may contribute to a better understanding of regional immune responses to circulating influenza strains. This could indirectly support future refinements in vaccine strain selection and targeted public health interventions (e.g., prioritizing vaccination in high-risk groups with distinct inflammatory signatures), pending validation with larger cohorts and protein-level data.

## 2. Materials and Methods

### 2.1. Study Population

A total of 380 nasopharyngeal aspirates (NPAs) were collected from patients admitted to King Khalid University Hospital (KKUH) in Riyadh, Saudi Arabia, during the winter seasons (2020/21, 2021/22, and 2022/23). Samples were collected from patients residing in Riyadh at the time of admission, primarily Saudis and resident expatriates, excluding transient visitors such as pilgrims. Inclusion criteria included patients with fever (>38 °C) and respiratory symptoms, including cough, sore throat, and runny nose. Sampling was performed consecutively during peak influenza periods (November–March) to capture the full spectrum of eligible cases. Exclusion criteria included known co-infections with other respiratory viruses (e.g., RSV, SARS-CoV-2, or other confirmed pathogens via routine hospital testing), immunocompromised status (e.g., active chemotherapy, HIV, or high-dose corticosteroids), chronic respiratory diseases that could confound inflammatory profiles (e.g., severe COPD or cystic fibrosis), and patients who declined consent. Essential epidemiological data collected included patient ages, stopover sites, and sample collection dates. The study was conducted in accordance with the Declaration of Helsinki and approved by the Research Ethics Committee at King Saud University in Riyadh, Saudi Arabia (Institutional Review Board No. 22/0957/IRB, approved 27 November 2022).

### 2.2. Virus Detection

Viral RNA was extracted from the NPAs using the QIAamp Viral RNA Extraction Kit (Qiagen, Hilden, Germany, Cat. No. 52906) according to the manufacturer’s instructions. IAV was detected using universal primers targeting the *M* gene (Table 1), with the One-Step Ahead RT-PCR Kit containing Taq High Fidelity DNA Polymerase (Qiagen, Hilden, Germany, Cat. No. 220213). Subtyping for A/H1N1 and A/H3N2 was performed using specific primers (Table 1). IBV was detected using primers targeting the *NS-2* gene (Table 1). All RT-PCR reactions were performed on a GeneAmp 9700 thermal cycler (Applied Biosystems, Foster City, CA, USA) under standardized conditions: reverse transcription at 50 °C for 30 min, initial denaturation at 95 °C for 15 min, followed by 40 cycles of denaturation at 94 °C for 30 s, annealing at 55 °C for 90 s, and extension at 72 °C for 90 s, with a final extension at 72 °C for 10 min. PCR products were visualized on a 1% ethidium bromide-stained agarose gel and compared to a 100 bp DNA ladder (Qiagen, Hilden, Germany).

### 2.3. Quantitative Real-Time PCR

Total RNA from the NPAs was extracted from clinical samples using the RNeasy Mini Kit (Qiagen, Hilden, Germany) according to the manufacturer’s instructions. mRNA expression of cytokine and chemokine in NPAs was quantified via one-step quantitative RT-qPCR using the RT^2^ SYBR Green/ROX qPCR Master Mix (Qiagen, Hilden, Germany). SYBR Green chemistry was selected for its cost-effectiveness and established use in similar cytokine profiling studies [35]. To ensure specificity, all reactions included melting curve analysis confirming single-peak amplicons (absence of primer dimers or non-specific products) and no-template controls. Amplification efficiencies were determined from standard curves (range 90–110% for all primer pairs, with R^2^ > 0.98). Primer sequences and validation details (including melt curve examples) are provided in our prior publication [35], which used identical primer sets for respiratory virus cytokine profiling. Reactions were performed in a 7500 Fast Real-Time PCR System (Applied Biosystems, Foster City, CA, USA) with the following conditions: reverse transcription at 37 °C for 15 min, initial denaturation at 95 °C for 10 min, followed by 40 cycles of annealing at 60 °C for 30 s and extension at 72 °C for 30 s. Data were expressed as mean fold changes ± standard deviation (SD) from three independent amplifications. Two separate qPCR runs were performed: one comparative run including all IAV-positive (*n* = 65) and IBV-positive (*n* = 20) samples together with non-infected controls for direct IAV vs. IBV profiling; a subsequent run was conducted on IAV-positive samples only to enable detailed subtype (A/H1N1 vs. A/H3N2) comparisons. All runs used the same primer sets, master mix, and normalization to *GAPDH*, but minor batch effects may contribute to small differences between the pooled IAV values. 

### 2.4. Analysis of Real-Time PCR Array Data and Visualization of Inflammatory Profiles

All arrays were run under identical conditions, with Ct values determined using a constant baseline threshold. Samples were analyzed in duplicate across at least three independent experiments. Melting curve analysis confirmed amplification specificity and absence of primer dimers. Results were calculated using the 2^−ΔΔCt^ method [36], with ΔCt values normalized to GAPDH as the housekeeping gene. Fold changes were referenced to non-infected controls.

Mean fold-change values for the 17 profiled inflammatory mediators (*IFN-α*, *TNF-α*, *IL-1α*, *IL-2*, *IL-4*, *IL-6*, *IL-8*, *IL-10*, *IL-13*, *IL-17*, *IL-22*, *IL-33*, *G-CSF*, *CCL-2*, *CCL-3*, *CCL-4*, *CCL-5*) across infection groups (IAV pooled, IBV) and IAV subtypes (A/H1N1, A/H3N2) were prepared as a tab-delimited text matrix with mediators as rows and groups as columns. To visualize relative expression patterns, the matrix was uploaded to the Expression Heat Map tool at Heatmapper (https://heatmapper.ca/expression/accessed on 10 January 2026). Column z-score normalization was applied to standardize values across groups and emphasize patterns over absolute fold differences. Hierarchical clustering was performed on both rows and columns using Euclidean distance and average linkage. A red–green color gradient was selected (red indicating upregulation, green downregulation). The processed heatmap was exported as high-resolution PNG and SVG files for further analysis. The heatmap was generated with column z-score normalization to standardize values across groups. Hierarchical clustering was performed on both rows and columns using Euclidean distance and average linkage. Sample sizes were: IAV pooled (*n* = 65), IBV (*n* = 20), A/H1N1 (*n* = 30), and A/H3N2 (*n* = 35).

To investigate potential regulatory interconnections among the profiled mediators, a protein–protein interaction network was generated using GeneMANIA (https://genemania.org/, application version 3.6.0) for Homo sapiens. The 17 query genes corresponding to the mediators were entered as the input list. Network weighting was assigned automatically based on the query genes. The tool integrated multiple evidence types, including co-expression, shared protein domains, physical interactions, genetic interactions, predicted interactions, co-localization, and pathway linkages from databases such as BioGRID, INTERPRO, NCI-Nature Pathways, and others. The resulting network, including query genes as core nodes and automatically added associated genes ranked by relevance, was visualized using the default layout with edge colors indicating interaction types. The network was exported as PNG and SVG files for further analysis.

### 2.5. Statistical Analysis

All experiments were performed in triplicate, with results expressed as mean ± SD. Analyses were conducted using GraphPad Prism software (version 5.0; GraphPad Software, San Diego, CA, USA). Non-parametric one-way ANOVA with post hoc Dunnett’s test was used for multiple comparisons of cytokine/chemokine mRNA fold-change data across infection groups (IAV pooled vs. IBV) and IAV subtypes (A/H1N1 vs. A/H3N2). Independent samples *t*-tests were applied for pairwise comparisons of cytokine/chemokine mRNA expression between groups (e.g., IAV vs. IBV, A/H1N1 vs. A/H3N2). Categorical data (prevalence rates by season, gender, and age group) were presented as frequencies and percentages and compared using the chi-square test. Non-Gaussian variables were reported as medians where applicable. A *p*-value < 0.05 was considered statistically significant. To control multiple comparisons across the 17 mediators, all *p*-values were adjusted using the Benjamini–Hochberg false discovery rate (FDR) method; FDR-adjusted *p*-values < 0.05 were considered significant.

## 3. Results

### 3.1. Demographic Analysis and IAV/IBV Prevalence

IAV subtype (A/H3N2) was the most frequent (9.21%, *n* = 35), followed by A/H1N1 pdm09 (7.89%, *n* = 30) and IBV (5.26%, *n* = 20). Overall IAV positivity was 17.11% (*n* = 65). Positivity rates increased across seasons, from 11.67% in 2020/21 to 26.15% in 2022/23. Males showed higher IAV prevalence (25.88%) than females (10.00%; *p* < 0.05), driven by both subtypes (A/H1N1: 9.41% vs. 6.67%; A/H3N2: 16.47% vs. 3.33%). Conversely, IBV was more common in females (6.67%) than males (3.53%). Patients were categorized into age groups: 0–4, 5–14, 15–64, and ≥65 years [21]. Patients were categorized into these age groups following standard epidemiological classifications used in influenza studies to reflect different risk levels in young children, school-aged children, adults, and the elderly. The 0–4-year group had the highest IAV positivity (28.42%; *p* < 0.05 vs. other groups), with A/H1N1 at 13.68% and A/H3N2 at 14.75%. IBV was most prevalent in the 5–14-year group (10.91%). Detailed distributions are shown in Table 2.

**Table 2:** Sample distribution across epidemic seasons (winters 2020–2023), gender, and age groups.

Category	No. of Samples N (%)	Positive for IAV n (%)	Positive for A/H1N1 n (%)	Positive for A/H3N2 n (%)	Positive for IBV n (%)
Total	380 (100)	65 (17.11)	30 (7.89)	35 (9.21)	20 (5.26)
Season					
2020/21	120 (31.57)	14 (11.67)	6 (5.00)	8 (6.67)	5 (4.16)
2021/22	130 (34.21)	17 (13.08)	6 (4.62)	11 (8.46)	7 (5.38)
2022/23	130 (34.21)	34 (26.15)	18 (13.85)	16 (12.31)	8 (6.15)
Gender					
Male	170 (44.74)	44 (25.88) ^a^	16 (9.41) ^a^	28 (16.47) ^a^	6 (3.53)
Female	210 (55.26)	21 (10.00)	14 (6.67)	7 (3.33)	14 (6.67)
Age (Years)					
0–4	95 (25.00)	27 (28.42) ^b^	13 (13.68) ^b^	14 (14.74) ^b^	3 (3.16)
5–14	110 (28.95)	19 (17.27)	7 (6.36)	12 (10.91)	12 (10.91)
15–64	120 (31.58)	9 (7.50)	4 (3.33)	5 (4.17)	4 (3.33)
≥65	55 (14.47)	10 (18.18)	6 (10.91)	4 (7.27)	1 (1.82)

Data are displayed as percentages (%). Comparisons between groups were performed using Chi-square test. ᵃ Significantly higher (*p* < 0.05) than females. ᵇ Significantly higher (*p* < 0.05) than age groups 5–14, 15–64, and ≥65 years.

### 3.2. Cytokine and Chemokine Profiles

**Figure 1:**
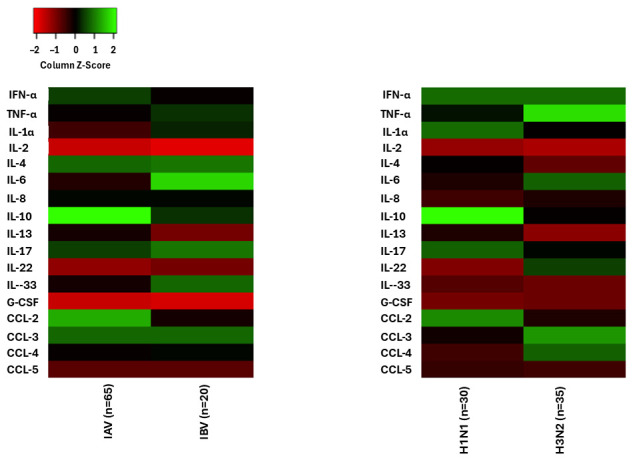
Heatmap of column z-scores for influenza A virus (IAV, *n* = 65) and influenza B virus (IBV, *n* = 20) in the left panel, and for A/H1N1 (*n* = 30) and A/H3N2 (*n* = 35) subtypes in the right panel, based on mRNA expression of 17 inflammatory mediators. The color scale represents standardized column z-scores (−2 to 2), with red indicating negative deviations (below the column mean) and green indicating positive deviations (above the column mean).

IAV patients exhibited significantly higher mRNA expression of *IFN-α* (*p* = 0.002), *IL-10* (*p* = 0.006), *IL-13* (*p* = 0.018), and *CCL-2* (*p* = 0.001) compared to IBV patients, whereas IBV patients showed higher *IL-1α* (*p* = 0.001), *IL-6* (*p* = 0.005), and *IL-33* (*p* = 0.021) (Table 3; Figure 1). This differential expression pattern is clearly visualized in the heatmap (Figure 1), where column z-scores highlight elevated *IFN-α*, *IL-10*, *IL-13*, and *CCL-2* in the IAV group (*n* = 65) relative to IBV (*n* = 20), contrasted with stronger upregulation of *IL-1α*, *IL-6*, and *IL-33* in IBV. Notably, the mean fold-change values for IAV in Table 3 (derived from the direct IAV–IBV comparative qPCR run) differ slightly from those that would be obtained by weighting the subtype-specific values in Table 4; this is attributable to the use of separate qPCR runs for the pooled IAV–IBV comparison versus the A/H1N1–A/H3N2 subtype analysis.

Within IAV infections, A/H1N1 patients (*n* = 30) displayed significantly higher mRNA expression of *IL-4* (*p* = 0.001), *IL-10* (*p* < 0.001), *IL-13* (*p* = 0.017), *IL-17* (*p* = 0.002), and *IL-1α* (*p* = 0.001) than A/H3N2 patients (*n* = 35), whereas A/H3N2 patients had significantly higher *TNF-α* (*p* = 0.005), *IL-6* (*p* = 0.008), *IL-22* (*p* = 0.006), *CCL-3* (*p* = 0.002), and *CCL-4* (*p* < 0.001) than A/H1N1 patients (Table 4; Figure 1). The heatmap further illustrates these subtype distinctions, with the right panel showing prominent green (upregulated) signals for *IL-4*, *IL-10*, *IL-13*, *IL-17*, and *IL-1α* in A/H1N1, contrasted with stronger red (upregulated) signals for *TNF-α*, *IL-6*, *IL-22*, *CCL-3*, and *CCL-4* in A/H3N2, reinforcing the distinct inflammatory signatures associated with each IAV subtype.

**Table 3:** Comparison of cytokine and chemokine profiles between IAV and IBV patients.

Mediator	IAV (*n* = 65)	IBV (*n* = 20)	*p*-Value
*IFN-α*	5.32 ± 1.34	4.32 ± 2.24	0.002
*TNF-α*	4.22 ± 1.24	4.92 ± 2.14	0.212
*IL-1α*	3.33 ± 1.38	4.72 ± 2.66	0.001
*IL-2*	1.32 ± 1.34	1.52 ± 0.14	0.626
*IL-4*	5.92 ± 1.12	5.72 ± 2.11	0.445
*IL-6*	3.82 ± 2.94	7.02 ± 1.91	0.005
*IL-8*	4.36 ± 1.55	4.45 ± 1.81	0.113
*IL-10*	8.32 ± 1.77	4.89 ± 2.38	0.006
*IL-13*	3.96 ± 1.36	2.92 ± 0.94	0.018
*IL-17*	5.22 ± 0.39	5.77 ± 2.84	0.356
*IL-22*	2.12 ± 1.34	2.88 ± 0.14	0.522
*IL-33*	3.92 ± 0.94	5.66 ± 1.34	0.021
*G-CSF*	1.32 ± 1.24	1.66 ± 0.38	0.133
*CCL-2*	6.92 ± 1.88	4.12 ± 1.14	0.001
*CCL-3*	5.98 ± 2.64	5.52 ± 2.67	0.551
*CCL-4*	4.22 ± 1.04	4.37 ± 2.37	0.737
*CCL-5*	3.02 ± 1.24	3.32 ± 0.39	0.547

Note: Values are means ± SD from triplicates. Statistical analysis used Student’s *t*-test; *p* < 0.05 considered significant. *p*-values are FDR-adjusted using the Benjamini–Hochberg method.

**Table 4:** Comparison of cytokine and chemokine profiles between A/H1N1 and A/H3N2 patients.

Mediator	A/H1N1 (*n* = 30)	A/H3N2 (*n* = 35)	*p*-Value
*IFN-α*	6.42 ± 0.99	6.56 ± 2.11	0.467
*TNF-α*	4.67 ± 1.78	8.94 ± 2.09	0.005
*IL-1α*	6.33 ± 2.08	4.46 ± 1.66	0.001
*IL-2*	1.62 ± 1.35	1.22 ± 0.13	0.212
*IL-4*	4.24 ± 1.62	2.72 ± 2.91	0.001
*IL-6*	3.82 ± 2.94	6.42 ± 1.61	0.008
*IL-8*	3.35 ± 2.05	4.11 ± 1.31	0.224
*IL-10*	9.36 ± 1.54	4.49 ± 2.58	<0.001
*IL-13*	3.94 ± 1.35	1.92 ± 0.54	0.017
*IL-17*	6.26 ± 0.39	4.77 ± 1.85	0.002
*IL-22*	2.12 ± 1.34	5.85 ± 1.14	0.006
*IL-33*	2.92 ± 0.54	2.66 ± 0.34	0.139
*G-CSF*	2.33 ± 1.24	2.62 ± 1.38	0.159
*CCL-2*	6.92 ± 1.88	4.12 ± 1.14	0.198
*CCL-3*	4.18 ± 2.14	7.52 ± 2.17	0.002
*CCL-4*	3.22 ± 1.24	6.37 ± 2.17	<0.001
*CCL-5*	3.42 ± 1.25	3.34 ± 1.39	0.119

Note: Values are means ± SD from triplicates. Statistical analysis used Student’s *t*-test; *p* < 0.05 considered significant. *p*-values are FDR-adjusted using the Benjamini–Hochberg method.

### 3.3. Functional Interaction Network Among Profiled Cytokines and Chemokines

To further elucidate the interconnected regulatory dynamics among the 17 inflammatory mediators profiled in this study (*IFN-α*, *TNF-α*, *IL-1α*, *IL-2*, *IL-4*, *IL-6*, *IL-8*, *IL-10*, *IL-13*, *IL-17*, *IL-22*, *IL-33*, *G-CSF*, *CCL-2*, *CCL-3*, *CCL-4*, and *CCL-5*), a comprehensive interaction network was constructed using GeneMANIA (version 3.6.0, data update August 2021), which integrated diverse data sources including physical interactions, co-expression, shared protein domains, genetic interactions, predicted associations, co-localization, and pathway linkages from over 300 high-throughput studies and databases such as BioGRID, INTERPRO, IRefIndex, and NCI-Nature Pathways. The network was weighted automatically based on the query genes and revealed a densely interconnected graph with the original 17 cytokines as core nodes, augmented by 17 additional related genes (*IL20*, *IL19*, *IFNA6*, *IL26*, *IFNA13*, *CCL8*, *IFNG*, *IL24*, *CCL15*, *IFNA14*, *IFNA17*, *CXCL2*, *IL11*, *CSF2*, *CCL4L2*, *IL1B*, and *LIF*) ranked by relevance, forming distinct clusters that highlight functional groupings such as type I interferons (e.g., *IFNA1*, *IFNA6*, *IFNA13*, *IFNA14*, *IFNA17*) tightly linked via co-expression and shared domains, pro-inflammatory interleukins and chemokines (e.g., *IL-6*, *TNF*, *IL-1A*, *IL-1B*, *CCL2*, *CCL3*, *CCL4*, *CCL5*, *CCL8*, *CXCL2*, *CXCL8*) interconnected predominantly through physical interactions and pathways indicative of innate immune activation and chemotaxis, and anti-inflammatory or Th2/Th17-associated cytokines (e.g., IL-4, IL-10, IL-13, IL-17A, IL-22, IL-33) bridged by genetic and predicted interactions suggesting regulatory feedback loops.

**Figure 2:**
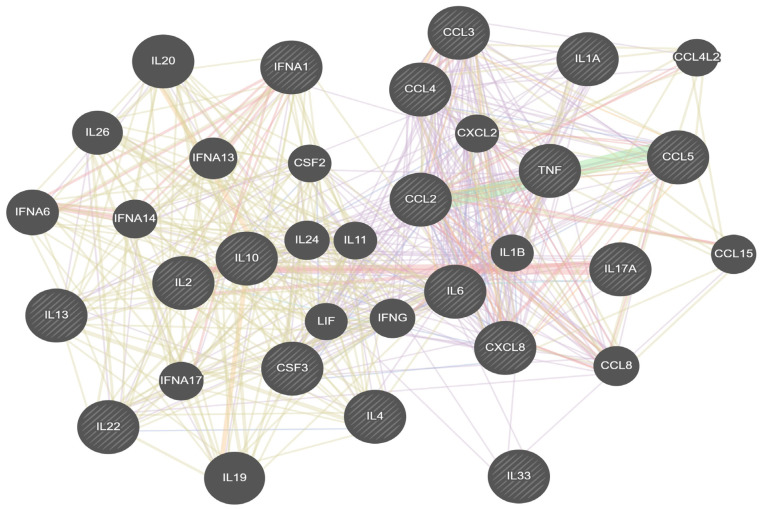
Protein–protein interaction network of the 17 inflammatory mediators profiled in influenza A and B infections, generated using GeneMANIA (v3.6.0; database update 13 August 2021) for Homo sapiens. The network includes the query genes as core nodes, expanded by 17 top-ranked associated genes (e.g., *IL20*, *IL19*, *IFNA6*, *IL26*, *IFNA13*, *CCL8*, *IFNG*, *IL24*, *CCL15*, *IL1B*, *LIF*). Edge colors represent interaction types: co-expression (pink, 45.92%), shared protein domains (beige, 19.14%), physical interactions (red, 17.76%), genetic interactions (green, 9.96%), predicted interactions (orange, 3.06%), co-localization (light blue, 3.05%), and pathway linkages (purple, 1.11%), sourced from BioGRID, INTERPRO, NCI-Nature Pathways, and others. The layout highlights functional clusters (type I interferons, pro-inflammatory chemokines, Th2/Th17 cytokines) that align with subtype-specific inflammatory profiles observed in the study.

Notably, co-expression accounted for the largest proportion of edges (45.92%, primarily from immune response datasets like Boldrick-Relman-2002 on bacterial innate immunity and Arijs-Rutgeerts-2009 on IBD mucosal inflammation), followed by shared protein domains (19.14% from INTERPRO, reflecting structural similarities in cytokine families), physical interactions (17.76%, e.g., from BioPlex networks in Huttlin-Gygi-2015 and Huttlin-Harper-2017), genetic interactions (9.96% from small-scale BioGRID studies), predicted associations (3.06% from functional human interactome models like Wu-Stein-2010), co-localization (3.05%, e.g., from splicing microarray data in Johnson-Shoemaker-2003), and pathway linkages (1.11% from NCI-Nature, emphasizing cytokine-receptor signaling cascades); this network topology (Figure 2) underscores the subtype-specific inflammatory signatures observed, with A/H3N2-associated mediators (e.g., *TNF-α*, *IL-6*, *CCL-3*, *CCL-4*) centrally positioned in pro-inflammatory hubs potentially contributing to amplified inflammation, while A/H1N1-linked ones (e.g., *IFN-α*, *IL-10*, *IL-13*) cluster with antiviral and regulatory nodes. The IBV-associated mediators *IL-1α* and *IL-33* are themselves alarmins that contribute to innate immune and epithelial repair pathways.

## 4. Discussion

The findings from this study offer descriptive insights into mRNA expression patterns and molecular epidemiology of IAV and IBV viruses in a Saudi Arabian cohort, specifically from Riyadh during the 2020–2023 winter seasons. A total of 380 NPAs were analyzed, revealing an overall influenza positivity rate of 22.37%, with IAV accounting for 17.11% (65 cases) and IBV for 5.26% (20 cases). Within A/H3N2 was the predominant subtype at 9.21% (35 cases), followed by A/H1N1 at 7.89% (30 cases). This distribution aligns with global trends where A/H3N2 often dominates seasonal epidemics due to its higher mutation rate and antigenic drift, as evidenced by recent phylogenetic analyses showing clade shifts from 6B.1 in earlier years to 5a.2a post-2020 [36,37,38,39]. In Saudi Arabia, similar patterns have been reported, with A/H3N2 comprising nearly half of IAV cases in Riyadh from 2014 to 2020, clustering in clades 3c.2a1b.1 and 3c.2a1 [8,11,16,40]. The observed increase in positivity from 11.67% in 2020/21 to 26.15% in 2022/23 likely reflects the relaxation of COVID-19 restrictions, which suppressed influenza circulation through measures like masking and social distancing [40,41]. Notably, the absence of B/Yamagata lineage detections post-2020 in global surveillance, including limited reports in Saudi Arabia, suggests its potential extinction, prompting discussions on quadrivalent vaccine reformulation to trivalent [28]. This study’s seasonal focus (November-March) captures peak transmission, consistent with Middle Eastern patterns influenced by cooler weather and increased indoor activities [40,42,43].

Demographic analysis revealed significant gender and age disparities in infection rates. Males exhibited higher IAV positivity (25.88%) compared to females (10.00%), particularly for A/H3N2 (16.47% vs. 3.33%), with statistical significance. This male predominance may stem from behavioral factors, such as greater occupational exposure or delayed healthcare seeking, rather than inherent immunological differences, as supported by epidemiological data from Saudi viral disease reports [40,42]. Conversely, IBV was more prevalent in females (6.67% vs. 3.53%), aligning with observations that IBV lineages like Victoria affect diverse demographics but show female bias in some cohorts [25]. Age stratification indicated the highest IAV rates in the 0–4 year group (28.42%), significantly exceeding other categories, which corroborates pediatric vulnerability due to immature immunity and higher exposure in daycare settings [12,44]. IBV peaked in the 5–14 year group (10.91%), consistent with school-aged transmission hotspots noted in US and European data, where IBV accounts for 22–44% of pediatric influenza deaths [25,45]. Elderly (≥65 years) showed moderate rates (18.18% for IAV), potentially mitigated by vaccination uptake, though comorbidities elevate severity risks [31,46]. These patterns underscore the need for targeted vaccination campaigns in Saudi Arabia, focusing on children and males, to reduce community transmission [11,16,30].

The cytokine and chemokine profiles elucidated distinct inflammatory signatures between IAV and IBV infections, highlighting pathogen-specific immune modulation. IAV patients exhibited significantly higher mRNA expression of *IFN-α* (5.32 ± 1.34 vs. 4.32 ± 2.24), *IL-10* (8.32 ± 1.77 vs. 4.89 ± 2.38), *IL-13* (3.96 ± 1.36 vs. 2.92 ± 0.94), and *CCL-2* (6.92 ± 1.88 vs. 4.12 ± 1.14) compared to IBV. These elevations suggest a robust Th2 and regulatory response in IAV, promoting anti-inflammatory balance but potentially contributing to immunopathology if unchecked [19,22,23,47]. In contrast, IBV induced higher *IL-1α* (4.72 ± 2.66 vs. 3.33 ± 1.38), *IL-6* (7.02 ± 1.91 vs. 3.82 ± 2.94), and *IL-33* (5.66 ± 1.34 vs. 3.92 ± 0.94), indicative of stronger inflammasome activation and epithelial repair signaling, which may explain IBV’s association with milder but prolonged symptoms [28,48,49]. These differences could arise from viral protein interactions; for instance, IAV’s NS1 protein more effectively suppresses IFN pathways than IBV’s counterpart, leading to compensatory cytokine surges [18,50,51]. Bibliometric analyses of influenza-cytokine research emphasize hotspots like *IL-6* and *TNF-α* in severity prediction, with over 1000 publications from 2013 to 2023 focusing on these mediators [24,52]. Recent reviews further underscore the central role of IL-6/TNF-α/IL-1 pathways in viral respiratory cytokine dysregulation and hyperinflammation across influenza infections [21,26,52,53,54]. In this cohort, no significant differences were noted in *TNF-α*, *IL-2*, or other CCLs between IAV and IBV, suggesting shared pathways in chemotaxis and granulocyte recruitment [19,23,26].

The GeneMANIA interaction network provides a broader regulatory context for these observations, revealing functional clusters that align with the subtype-specific inflammatory signatures. Pro-inflammatory mediators elevated in A/H3N2 (e.g., *TNF-α*, *IL-6*, *CCL-3/4*) occupy central hubs, supporting their role in amplifying hyperinflammation and cytokine storms, consistent with A/H3N2’s association with greater disease severity in seasonal epidemics. In contrast, antiviral/regulatory factors prominent in A/H1N1 and overall IAV (e.g., *IFN-α*, *IL-10*, *IL-13*) cluster with type I interferon pathways, reflecting balanced responses that may limit excessive inflammation. The IBV-associated mediators *IL-1α* and *IL-33* are themselves alarmins that can contribute to inflammasome priming and amplification of innate inflammatory responses (in contrast to *IL-1β*, which is the primary mature cytokine cleaved and secreted by activated inflammasomes). *IL-1β* was not included in the profiled mediator panel, as our focus was on a selected set of 17 cytokines/chemokines based on prior relevance in respiratory viral infections [35]. Future studies could expand the panel to include *IL-1β* and other inflammasome-specific markers for a more complete assessment of inflammasome involvement. These interconnections suggest coordinated regulatory loops that could drive differential outcomes across subtypes. Recent studies reinforce this view, with [22] linking elevated *IL-6* and *TNF-α* to severe influenza outcomes and systemic inflammation in hospitalized patients, [26] emphasizing IL-6/TNF-α/IL-1 pathways in cytokine storm pathogenesis across viral infections including influenza, [52] demonstrating IL-6’s pivotal contribution to IAV-driven hyperinflammation in severe cases, and [44] describing heightened IL-6/TNF-α/IFN responses in the unprecedented 2024/2025 influenza epidemic, underscoring subtype-specific amplification. No enriched functions were identified (N/A), likely due to the focused query set, but the network topology offers a mechanistic framework for understanding why A/H3N2 infections may more readily escalate to severe pathology and support targeted interventions (e.g., *IL-6* pathway modulators) in high-risk cases. While useful for visualization and hypothesis generation, the network integrates multiple evidence types, including predicted associations, rather than solely experimentally validated interactions.

Integrating molecular surveillance data, this study detected IAV using universal primers and IBV via *NS-2* gene targeting, with phylogenetic alignment to vaccine strains indicating potential efficacy [8]. However, the presence of resistance mutations like S247N in some Saudi strains warrants monitoring for oseltamivir efficacy [8,32]. Metagenomic insights from similar studies show co-infections with bacteria like *Haemophilus influenzae*, which may exacerbate cytokine dysregulation [8,26]. Limitations of this study include its single-center, hospital-based design, which may introduce selection bias toward more severe or symptomatic cases, the absence of clinical severity scoring, and the lack of viral load quantification to correlate with mRNA expression patterns. The sampling was restricted to the winter months (November–March), capturing only the peak influenza season in Saudi Arabia and thus limiting conclusions about year-round trends. The higher IAV positivity observed in the 0–4 year group may partly reflect increased healthcare-seeking behavior and testing frequency in young children with fever, in addition to potential biological susceptibility. Furthermore, cytokine/chemokine profiling was based solely on mRNA expression in nasopharyngeal aspirates rather than secreted protein concentrations; although mRNA levels often correlate with protein production during inflammatory responses, post-transcriptional regulation, protein stability, and secretion dynamics can lead to discrepancies. The lack of longitudinal follow-up also restricts insights into disease progression. Future research should incorporate whole-genome sequencing for all isolates, assess microbiota-cytokine interactions (as alpha diversity varies with clades), and include protein quantification (e.g., via ELISA or multiplex bead arrays) to provide a more comprehensive view of mediator activity.

## 5. Conclusions

This study describes the molecular epidemiology and mRNA expression patterns of inflammatory mediators associated with IAV and IBV infections in Riyadh, Saudi Arabia, from 2020 to 2023. Key observations include the predominance of A/H3N2, an increase in prevalence following COVID-related restrictions, higher IAV positivity among males and young children, and distinct mRNA expression signatures visualized in the heatmap: higher mRNA expression of *IFN-α*, *IL-10*, *IL-13*, and *CCL-2* in IAV compared with elevated mRNA expression of *IL-1α*, *IL-6*, and *IL-33* in IBV. Subtype differences within IAV showed higher mRNA expression of *IL-4*, *IL-10*, *IL-13*, and *IL-17* in A/H1N1 and higher mRNA expression of *TNF-α*, *IL-6*, *IL-22*, *CCL-3*, and *CCL-4* in A/H3N2. The GeneMANIA interaction network provides a visual summary of potential regulatory connections among these mediators. These descriptive findings highlight the value of continued molecular surveillance in high-risk regions such as Saudi Arabia, where travel and mass gatherings may facilitate transmission. The results contribute to regional influenza data and may support future studies on vaccine strain selection and early intervention strategies.

## Figures and Tables

**Table 1:** Primers for IAV and IBV detection, typing, and sequencing.

Aim	Virus/Subtype	Primer Name	Sequence (5′–3′)	Amplicon Size (bp)	Ref
IAV detection (*M* gene)	IAV	M30F2/08	ATGAGYCTTYTAACCGAGGTCGAAACG	244	[33]
		M264R3/08	TGGACAAANCGTCTACGCTGCAG		
IBV detection (*NS-2* gene)	IBV	INFB-Univ-F	ATGGCCATCGGATCCTCAAC	238	[34]
		INFB-Univ-R	TGTCAGCTATTATGGAGCTG		
Typing primers	A/H1N1	(H1N1)-F	TGAGCTCAGTGTCATCATTTGA	174	[33]
		(A/H1N1)-R	TGCTGAGCTTTGGGTATGAA		
	A/H3N2	H3A1F6	AAGCAGGGGATAATTCTATTAACC	1127	
		H3A1R1	GTCTATCATTCCCTCCCAACCATT		
		NA-1F	GAGCAAAAGCAGGAGTAAAG	807	
		NA787R	TGACAATGTGCTAGTATGAAC		

## Data Availability

The original contributions presented in the study are included in the article, further inquiries can be directed to the corresponding author.
